# Posterior fixation of thoracolumbar burst fractures: Is it possible to protect one segment in the lumbar region?

**DOI:** 10.1007/s00590-013-1326-7

**Published:** 2013-10-05

**Authors:** Umut Canbek, Levent Karapınar, Ahmet İmerci, Ulaş Akgün, Mert Kumbaracı, Mustafa İncesu

**Affiliations:** 1 Department of Orthopaedics and Traumatology, Mugla Sıtkı Kocman University School of Medicine, Mugla, Turkey; 2 Department of Orthopaedics and Traumatology, Izmir Tepecik Education and Research Hospital, Izmir, Turkey; 3 Department of Orthopaedics and Traumatology, Erzurum Palandöken State Hospital, Osmangazi mah, Emniyet Sok. No. 35 Palandöken, 25000 Erzurum, Turkey

**Keywords:** Instrumentation, Thoracolumbar, Treatment

## Abstract

**Background:**

The treatment for thoracolumbar burst fractures is controversial. The aim of this retrospective study was to compare intermediate-segment (IS) and long-segment (LS) instrumentation in the treatment for these fractures.

**Methods:**

IS instrumentation was considered as pedicle fixation two levels above and one level below the fractured vertebra (infra-laminar hooks attached to lower vertebra with pedicle screws). LS instrumentation was done two levels above and two levels below the fractured vertebra. Among a total of 25 consecutive patients, Group 1 included ten patients treated by IS pedicle fixation, whereas Group 2 included fifteen patients treated by LS instrumentation.

**Results:**

The measurements of local kyphosis (*p* = 0.955), sagittal index (*p* = 0.128), anterior vertebral height compression (*p* = 0.230) and canal diameter expansion (*p* = 0.839) demonstrated similar improvement at the final follow-up between the two groups. However, there was a significant difference (*p* < 0.05) between Group 1 and Group 2 regarding clinical outcome [Hannover scoring system, Oswestry disability questionnaire and the range of motion of the lumbar region compared to neutral (0°)].

**Conclusions:**

The radiographic parameters were the same between the two groups. However, the clinical parameters demonstrated that IS instrumentation is a more effective management of thoracolumbar burst fractures.

## Introduction

Unstable fractures of the thoracolumbar spine often require internal fixation. Stabilisation of these injuries has many advantages, including early mobilisation of the patient and the potential for neurological improvement. The optimal treatment for these injuries is controversial.

The treatment modality that provides superior spinal canal restoration has not yet been conclusively identified [1, 2]. Various opinions exist regarding selection of the most effective surgical method to treat these fractures [3, 4]. The current consensus about treatment of this type of fracture is to fix the fewest number of vertebrae and to provide a safe fixation and neural canal decompression [4–6].

Short-segment posterior instrumentation is currently the most frequently used treatment modality [4, 6, 7]. Methods that support the anterior column or long-segment posterior instrumentation are applied to eliminate inadequate reduction, loss of reduction and inadequate correction of the spinal canal, all of which may be encountered following short-segment posterior instrumentation [4, 6, 8–11]. Long-segment posterior instrumentation involves immobilisation of more vertebrae although it provides a better fixation and better spinal canal remodelling. We consider that the negative aspects of both long- and short-segment posterior instrumentation may be eliminated with intermediate-segment instrumentation.

The aim of this study was to evaluate and compare the radiological and functional results between intermediate-segment (IS) instrumentation and long-segment (LS) instrumentation via a posterior approach in the treatment of thoracolumbar burst fractures.

## Materials and methods

### Subjects

In this study, 37 out of 50 patients who had been surgically treated by a single surgeon for thoracolumbar vertebra fractures between 2000 and 2009 were evaluated. Four patients were eliminated from the study because they developed neurological deficits, and eight patients were excluded from the study because they did not return for control visits, although they were invited. A total of 25 patients (14 females and 11 males) were retrospectively evaluated. Fourteen out of 25 patients had a type B fresh thoracolumbar burst fracture according to the Denis classification at L1, seven at T12 and four at L2 [12]. Injury involved all three columns. Patients were divided into two groups according to the type of surgery. Group 1 (IS instrumentation and fusion) included 10 patients (6 females and 4 males), and Group 2 (LS instrumentation and fusion) included 15 patients (8 females and 7 males). The mean ages of the patients were 32.3 years (range 17–52) and 36 years (range 19–50) in Group 1 and Group 2, respectively (Table 1).

**Table 1:** Descriptive characteristics of the research group

	Intermediate segment (IS)	Long segment (LS)
Age (years)	32.3 (17–52)	36 (19–50)
Follow-up period (months)	72.3 (31–102)	70.46 (5–104)
Gender (F/M)	6/4	8/7

### Surgical technique

Urgent decompression and vertebral alignment are required in the presence of neurological deficits. While some authors recommend the surgery when the patient is stable, some others prefer to wait for 4 days or longer until post-traumatic swelling decreases. All of our patients were operated on within the first 24 h after injury. A posterior approach to the thoracolumbar vertebrae is a well-described and accepted surgical procedure.

**Fig. 1:**
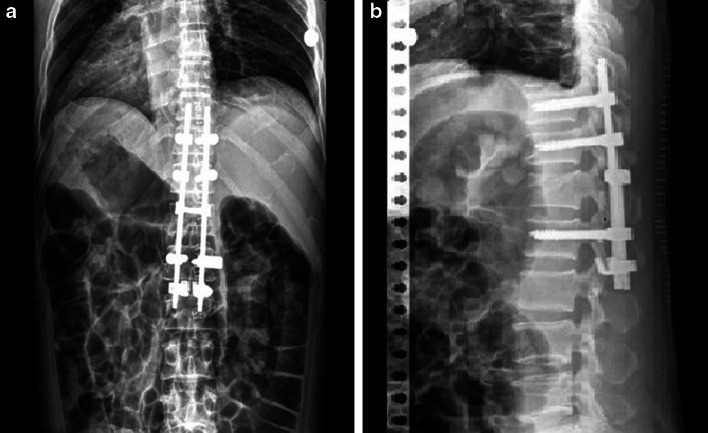
**a** Postero-anterior and **b** lateral radiographs of a patient with an L1 vertebra fracture who received IS instrumentation

**Fig. 2:**
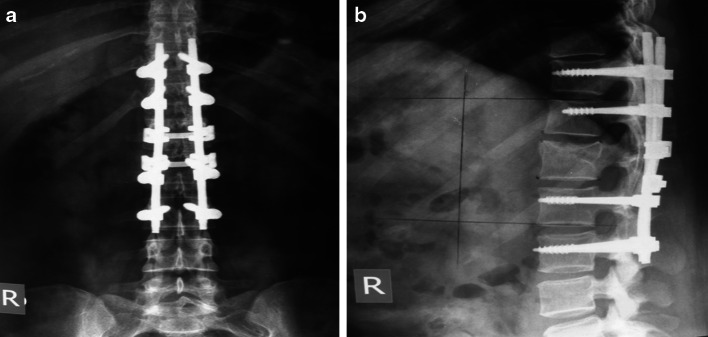
**a** Postero-anterior and **b** lateral radiographs of a patient with a T12 vertebra fracture who received LS instrumentation

Posterior instrumentation with fusion was used for vertebral surgery in our clinic. Adequate surgical exposure was performed via posterior midline incision. Pedicle screws were placed in accordance with surgical technique. If 2–1 instrumentation was performed, four trans-pedicular screws were inserted into the two vertebrae cranial to the fractured vertebra, two trans-pedicular screws were inserted into the one vertebra caudal to the fractured vertebra, and two laminar hooks were inserted into both laminae of the same vertebra on the caudal side (Fig. 1a, b). In the case of 2–2 instrumentation, a total of eight screws were inserted into two vertebrae cranial and caudal to the fractured vertebra (Fig. 2a, b). Considering the distraction that would be performed thereafter, two rods of appropriate size were chosen according to the instrumentation level. The rods were bound to each other using transverse connectors at a minimum of two levels, and strong stabilisation was provided sagittally, frontally and rotationally. Decompressive laminectomy was not performed.

**Fig. 3:**
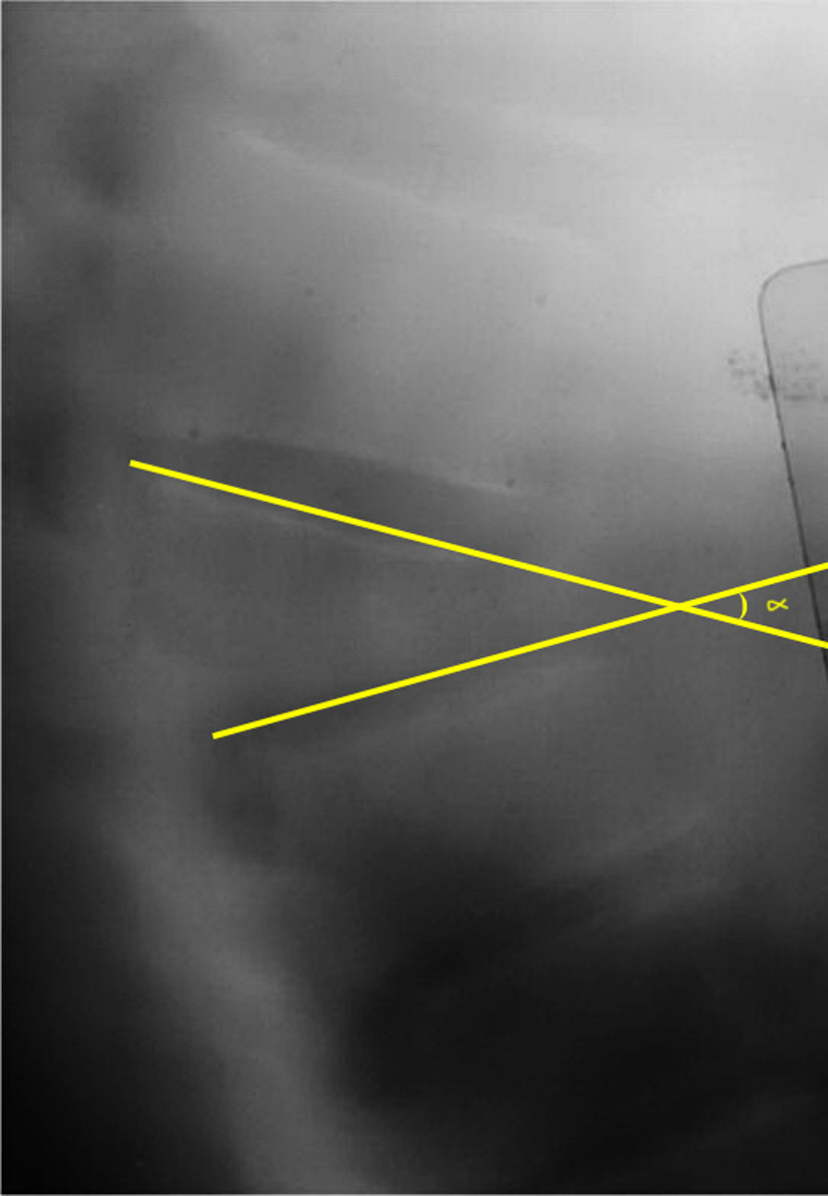
Sagittal index calculation in the lateral graphs

**Fig. 4:**
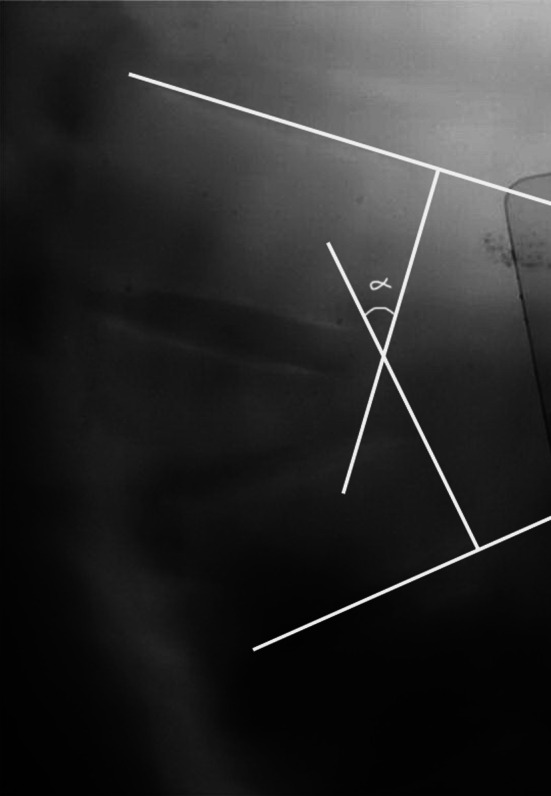
Calculation of Cobb’s angle in the lateral graphs

Sagittal deformities that developed after fracture were evaluated by determining anterior corpus height compression (ACHC), sagittal index and local kyphosis angle (LKA) with preoperative, post-operative and follow-up radiographs. Anterior corpus height decompression was calculated using the formula described by Mumford et al. [1]. The local kyphosis angle was determined by estimating the Cobb angle between the line passing through the uppermost endplate of the healthy vertebra and the line passing through the lowermost endplate of the lowest healthy vertebra (Fig. 3). The sagittal index was calculated with the measurement described by Farcy et al. [13] (Fig. 4). The correction of spinal canal narrowing due to retropulsed bone fragments was measured from axial CT sections in the immediate post-operative period long term. The mean spinal canal diameter at the levels of the upper and lower healthy vertebrae was measured, and the ideal diameter at the level of the fracture was calculated. The proportion of correction was calculated by dividing the current diameter by the ideal diameter.

Hannover vertebra scores [14], the Oswestry Disability Index [15] and the Visual Analogue Scale (VAS) [16] were filled in for the assessment of function and pain during the final visits of the patients. The joint range of motion was evaluated compared to neutral (0°). Variance analysis relied on repeated measurements to determine statistical significance in terms of the anterior vertebral height compression ratio, local kyphosis angle and sagittal index measurement values in the preoperative, post-operative and follow-up measurements in IS and LS instrumentation. A *p* value of <0.05 was accepted as statistically significant.

## Results

The mean duration between injury and surgery was 24 h. The patients were discharged from the hospital in an average of 6.3 days. The mean duration of follow-up was 72.3 months in the IS instrumentation group and 70.46 months in the LS instrumentation group.

While the mean sagittal index was 12.84 preoperatively in the IS instrumentation group, it was reduced to −1.01 post-operatively and then increased to 1.9 on final follow-up radiographs. The sagittal index measured 12.13 preoperatively, −1.01 post-operatively and 1.54 on follow-up radiographs in the LS instrumentation group. Although there was no significant difference between the two groups in terms of sagittal index, preoperative, post-operative and late post-operative follow-up results significantly differed between the two groups (*p* = 0.128). The mean anterior corpus height compression decreased from 36.10 % preoperatively to 12.10 % post-operatively and then increased to 18.80 % at follow-up in the IS instrumentation group. The mean anterior corpus height compression decreased from 34.45 % preoperatively to 11.33 % post-operatively and then increased to 15.63 % on follow-up radiographs in the LS instrumentation group. While the mean LKA measured 15.30° preoperatively in the IS instrumentation group and 9.62° in the LS instrumentation group, it was reduced to 1.20° in the IS instrumentation group and 0.30° in the LS instrumentation group post-operatively. On follow-up radiographs, the LKA measured 3.15° in both groups. Statistically significant differences were not found in ACHC or LKA, and the reductions in the degree of correction were also statistically insignificant (*p* = 0.230 for ACHC and *p* = 0.955 for LKA).

When the diameter of spinal measured preoperatively was compared to post-operative measurements in the long term, the diameter of the spinal canal in the long term post-operatively increased by 48.56 % in group 1 and by 47.01 % in group 2 patients compared to preoperative measurements. The difference between the IS and LS groups was not statistically significant (*p* = 0.839).

Hannover vertebra scoring and the Oswestry Disability Index for the assessment of functionality were completed for all patients. The mean Hannover vertebra score was 82 (70–85) in the LS instrumentation group and 62.1 (28–85) in the LS instrumentation group. The mean Oswestry disability score among the ten patients who underwent IS instrumentation was 14.4 % (12–24 %), and the mean Oswestry disability score of the 15 patients who underwent LS instrumentation was 29.2 % (12–58 %). In the IS instrumentation group, eight cases (80 %) reported low disability, two cases (20 %) reported moderate disability, and there were no reports on severe disability. In the LS instrumentation group, six cases (40 %) reported low disability, four cases (27 %) reported moderate disability, and five (33 %) reported severe disability. The difference in long-term Hannover vertebra scores of the patients in the IS and LS groups was statistically significant (*p* = 0.07).

Long-term range of motion was compared to neutral (0°). The mean flexion was 66.5°, the extension was 19.5°, the lateral flexion was 20.5°, and the rotation was 30.5° in the IS instrumentation group; in contrast, the mean flexion was 50.5°, the extension was 10.5°, the lateral flexion was 12.5°, and the rotation was 21.5° in the LS group. The difference between the groups was statistically significant (*p* < 0.05).

## Discussion

Trans-pedicular, short-segment fixation became popular after the introduction of trans-pedicular screws by Roy-Camille et al. [17] and development of the internal fixator by Dick et al. [18]. This approach includes pedicle screw fixation at one vertebra cranial to and one vertebra caudal to the fracture. Although this approach has several advantages, it has been associated with loss of surgical reduction and instrumentation failure.

Instrumentation failure occurs by either of primary mechanisms, implant failure or bony failure. Implant fatigue failure (screw bending or breakage) may occur weeks or months after the initial surgery and typically is observed in the strong, dense bone of young trauma patients [19]. Alternatively, bony failure results in the loosening, toggling or backing out of screws due to failure of the bone. This may occur early or late and is most often observed in older patients with weak osteoporotic bone [20, 21]. In the presence of a thoracolumbar burst fracture, some authors consider augmentation of the vertebral body by anterior column support with cancellous bone, cement, hydroxyapatite blocks grafting or bone graft substitute. Improvement in anterior load-bearing capacity has been reported with trans-pedicular bone grafting [22, 23].

In the past few years, a new perspective in the treatment of thoracolumbar trauma was offered with the development of minimally invasive techniques. Their objective is to minimise conventional approach morbidity, such as blood loss, iatrogenic muscle trauma, pain and functional deterioration. It has also been suggested as an adjunct to conventional posterior stabilisation, as it can minimise spinal levels requiring fusion [24]. This technique was shown to have less perioperative morbidity and reduced hospitalisation time. However, implant limitations, increased operative time and an overall more demanding surgical technique are drawbacks that have not permitted widespread acceptance of this method. Posterior percutaneous stabilisation can be used either as a stand-alone procedure or as an adjunct to minimally invasive anterior decompression [25]. Its concept is supported by the reported effectiveness of short-segment fixation and non-fusion techniques in thoracolumbar trauma [26]. In the past, implant characteristics posed several limitations to the technique. Precontoured rods often required the placement of terminal screws with a higher offset, resulting in implant prominence, especially in the thoracolumbar junction [27]. Moreover, insertion of the rods when polyaxial screws were used was technically challenging, while earlier systems did not permit reduction or distraction manoeuvres. Patient and occupational exposure to radiation remains an issue, requiring adequate surgeon education in order to minimise the need for fluoroscopy use [28]. Fusion with minimal posterior surgery is not possible, which necessitates late instrumentation removal [29].

Augmentation of the short-segment pedicle instrumentation (SSPI) construct with offset laminar hooks has been recommended as a means of preventing fixation failure in thoracolumbar fractures [9, 30]. The laminar hooks are thought to decrease the load transmitted between bone and the pedicle screws, thereby protecting both the screws and the bone.

Adding one level of fixation cranially will increase the construct stiffness. Although adding a single motion segment may artificially increase segmental stiffness, to some extent, the protective benefit to pedicle screw bending moments is real. Finally, the addition of a single motion segment cranial to the fracture does not affect the spinal range of motion or sacrifice the principles of SSPI because the thoracic segments are relatively immobile and do not influence the function of the lower lumbar spine [21, 31].

The clinical implications of our findings are that augmentation of an SSPI construct with sublaminar hooks results in a stronger construct while decreasing the bending moments on the screws that might predispose to device failure. Therefore, addition of sublaminar hooks may decrease the rate of clinical failures with SSPI for unstable thoracolumbar fractures while still maintaining the advantages of this system: minimal fusion length, three-column fixation and application through a posterior approach [31, 32].

Supplemental offset hooks significantly increased construct stiffness without sacrificing the principles of SSPI (limited lumbar fixation). Furthermore, offset hooks absorb some components of the construct strain, thereby reducing the bending moments transmitted to the screws and reducing the likelihood of screw failure in severely unstable fractures [31].

Some surgeons add pedicle screws at the fractured vertebrae, termed intermediate screws, as part of a short-segment construct. These screws theoretically may stiffen the construct by splitting the length of the rod that spans from the upper screw to the lower screw into two half-length parts. A shorter rod between two points of fixation will create increased stiffness, and the additional fixation point can theoretically decrease the motion at the metal–bone interface. Nevertheless, the true mechanical function of screws inserted into a fractured vertebra is unclear, as the pattern of a burst fracture involves comminution of the vertebral body and separation of the pedicles. To the authors’ knowledge, no study has been carried out to show the biomechanical effect of intermediate screws on the fixation of fractures. Dick et al. [18] evaluated the effect of adding screws at the intermediate level on the stiffness of a short-segment construct. Calf spine segments were used and destabilised anteriorly by dividing the disc annulus with a knife. They found that the addition of two intermediate screws increased stiffness in axial loading, flexion and torsion. Because no fracture model was created, no conclusions could be drawn as to the effect of these screws when fixed to a fractured vertebra [32].

In conclusion, the addition of supplemental hooks below the fractured vertebra with trans-pedicular screws inserted into the anterior cortex can result in less motion at the fractured segment within a short-segment construct. Short-segment fixation offers the advantage of preserving motion segments in the lumbar spine. The authors recommend the use of supplemental sublaminar hooks when posterior spinal fusion is indicated for the fixation of unstable thoracolumbar fractures.

In this study, no statistically significant difference was detected between the groups in terms of long-term functional and radiographic results. Methods supporting anterior column or LS instrumentation are applied to eliminate negative conditions such as the insufficient reduction, reduction loss and inadequate expansion of the spinal canal encountered after short-segment posterior instrumentation [6, 8, 9, 33]. Although LS instrumentation provides stronger fixation and superior spinal canal correction, it results in increased vertebral immobility, as it affects more segments. LS instrumentation has been shown to result in more mobile segment immobilisation and more dorsalgia in the future [34, 35]. Therefore, we recommend the IS instrumentation technique, a segment-preserving surgery that has not shown a difference from LS instrumentation either functionally or radiographically.

We recommend future research to compare short-segment and long-segment instrumentation for T12 vertebral fracture, L1 vertebral fracture and L2 vertebral fracture individually.
